# 2.5D Flexible Wind Sensor Using Differential Plate Capacitors

**DOI:** 10.3390/s21093101

**Published:** 2021-04-29

**Authors:** Yu Wan, Zhenxiang Yi

**Affiliations:** Key Laboratory of MEMS of the Ministry of Education, Southeast University, Nanjing 210096, China; 220191417@seu.edu.cn

**Keywords:** wind sensor, flexible, 2.5D, capacitors

## Abstract

In this paper, a novel 2.5-dimensional (2.5D) flexible wind sensor is proposed based on four differential plate capacitors. This design consists of a windward pillar, two electrode layers, and a support layer, which are all made of polydimethylsiloxane (PDMS) with different Young’s moduli. A 2 mm × 2 mm copper electrode array is located on each electrode layer, forming four parallel plate capacitors as the sensitive elements. The wind in the xy-plane tilts the windward pillar, decreasing two capacitances on the windward side and increasing two capacitances on the leeward side. The wind in the *z*-axis depresses the windward pillar, resulting in an increase of all four capacitances. Experiments demonstrate that this sensor can measure the wind speed up to 23.9 m/s and the wind direction over the full 360° range of the xy-plane. The sensitivities of wind speed are close to 4 fF·m^−1^·s and 3 fF·m^−1^·s in the xy-plane and *z*-axis, respectively.

## 1. Introduction

As a natural phenomenon, wind has a significant impact on our lives. The measurement of wind speed and direction plays a very important role in many fields, such as meteorological detection, transportation, agricultural production, and aerospace. According to the measurement principle, anemometers can be divided into four categories: mechanical anemometers, thermal anemometers, sonic anemometers, and pressure anemometers. The mechanical anemometer has been in use for more than 150 years [1]. It has several advantages, including a simple structure, low production cost, and a strong anti-interference ability, but it cannot meet people’s needs for portable devices due to its large size. Especially after the emergence of MEMS technology, the thermal anemometer began to attract the attention of researchers [2,3,4,5,6,7,8,9]. It has the advantages of miniaturization, high accuracy, and high sensitivity. However, the thermal anemometer requires high power consumption to form a temperature field. In this regard, the pressure anemometer uses some passive devices, such as resistors, inductors, and capacitors, to convert wind information into electrical signals, achieving extremely low power consumption [10,11,12,13,14,15,16,17]. However, in practice, the sensitive elements are directly exposed to the air and are susceptible to the interference of dust particles, leading to a decline in reliability. In addition, all of the anemometers above can only detect one-dimensional (1D) or two-dimensional (2D) wind speeds and direction measurements. However, in addition to the xy-plane, wind speed information in the *z*-axis is also needed in some areas, including unmanned aerial vehicle and indoor wind control systems. At present, the sonic anemometer is usually used for three-dimensional (3D) wind measurement, but it is sensitive to temperature changes, which can affect the measurement of the travel times along the sonic paths [1]. Moreover, it is expensive and difficult to miniaturize for use in portable devices.

Recently, flexible electronic devices have emerged [18,19]. The flexible material is deformed by external force, which can be characterized by capacitance variation. Therefore, in this paper, a novel flexible wind sensor based on differential plate capacitors is proposed. The sensor can measure the wind in the xy-plane and the negative *z*-axis and is described as a 2.5D (xy-plane and half of the *z*-axis) wind sensor due to the lack of the wind information in the positive *z*-axis. The electrodes of the capacitors are embedded in the substrate to avoid interference from the external environment directly, which can improve the reliability and lifespan of the device. Moreover, the wind speed sensor has the advantages of simple a fabrication process and no energy consumption. It is also worth mentioning that the sensitivity of the sensor can be improved by using four differential capacitors.

## 2. Design and Simulation

### 2.1. Design and Principle

**Figure 1:**
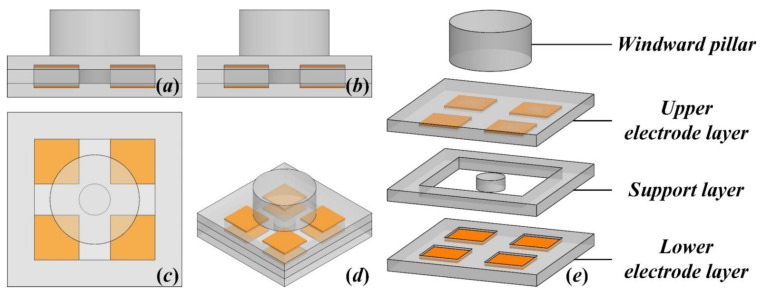
Schematic diagram of the proposed flexible wind sensor: (**a**) main view, (**b**) left view, (**c**) top view, (**d**) isometric view, and (**e**) hierarchical structure view.

The differential capacitive wind sensor proposed in this paper, as shown in Figure 1, consists of four polydimethylsiloxane (PDMS) layers, including a windward pillar, an upper electrode layer, a support layer, and a lower electrode layer. Among them, the radius and the thickness of the windward pillar are both designed to be 10 mm. The dimensions of the other layers are all about 40 mm × 40 mm × 3 mm. In particular, there is a micropillar with a radius of 3 mm in the center of the support layer, forming a lever structure with the windward pillar, which plays an important role in the measurement of wind speed and direction.

**Figure 2:**
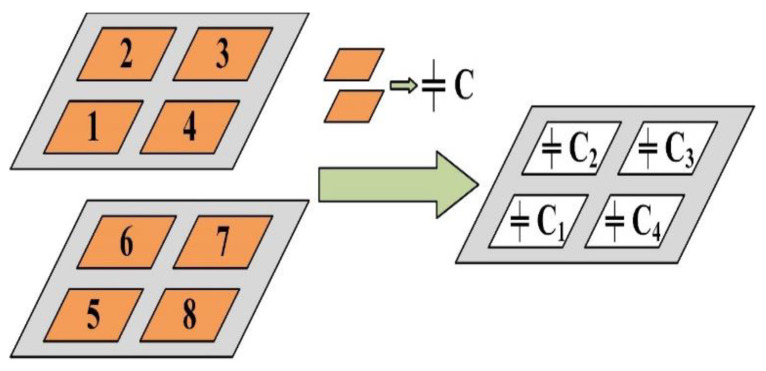
Schematic diagram of the four parallel plate capacitors.

There are 4 electrodes on each of the upper and lower electrode layers with a size of 10 mm × 10 mm, distributed in a 2 × 2 array. As shown in Figure 2, every two opposing electrodes (such as electrode 1 and electrode 5) form a parallel plate capacitor, so the sensor contains a 2 × 2 capacitor array. The capacitance value C of the parallel plate capacitor is defined by:

(1) $$C=\frac{\varepsilon S}{d}$$

where *ε* is the dielectric constant of the medium between the upper and lower electrodes, *S* is the area of the upper and lower electrodes facing each other, and *d* is the distance between the upper and lower electrodes. In Equation (1), fringing field capacitance is not considered. In this paper, *ε* and *S* are fixed, while d changes with the deformation of the upper electrode layer caused by the wind. Therefore, the speed and direction of the wind can be measured through the changes of the four differential capacitances.

Table 1 illustrates the measurement principle along the *x*, *y*, and *z* axes, respectively. On the *x*-axis, when the wind speed *v* > 0, the windward column tilts toward the positive *x*-axis. As a result, d1 and d2 increase, while d3 and d4 decrease, leading to a decrease in C_1_, C_2_ and an increase in C_3_, C_4_. Consequently, the wind speed can be extracted by the four capacitances measurements. Similarly, for the condition of *v* < 0, C_1_ and C_2_ increase while C_3_ and C_4_ decrease. The measurement principle on the *y*-axis is similar to that on the *x*-axis. If the wind blows the sensor from a certain angle to *x*-axis in the xy-plane, four capacitances vary with the wind direction according to the sine (or cosine) relationship.

For this proposed 2.5D wind sensor, in the *z*-axis, when the wind speed *v* < 0, the windward pillar and the upper electrode layer are forced to move downward by the wind, causing all four capacitances (C_1_, C_2_, C_3_ and C_4_) to increase. Obviously, the higher the wind speed, the greater the capacitances change.

Therefore, the wind sensor can transform the 2.5D wind field information into easily measurable capacitance information. The wind direction is characterized by the change in trend of capacitance and the wind speed is characterized by the change magnitude of capacitance.

### 2.2. Simulation by FEM

**Figure 3:**
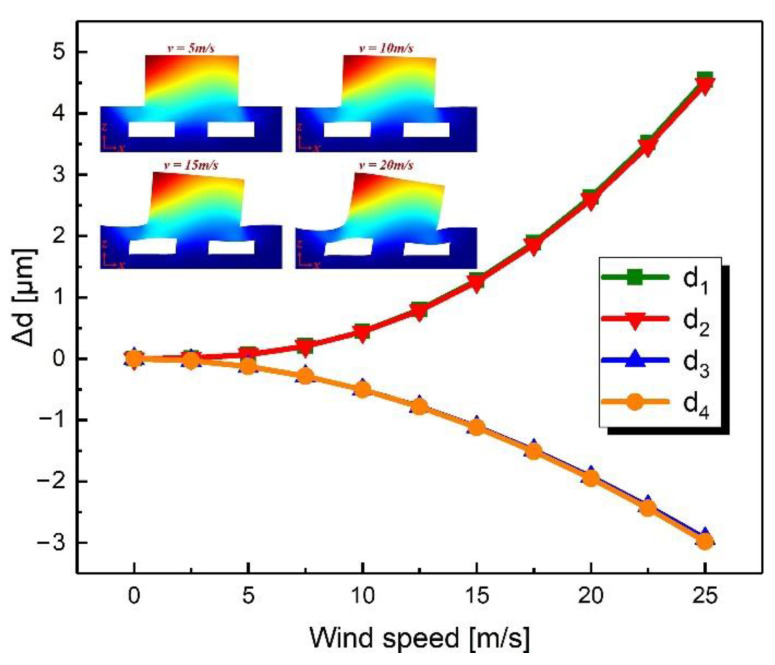
Simulated change in gap distances when the wind speed varied from 0 m/s to 25 m/s in the xy-plane with a fixed direction of 0°.

**Figure 4:**
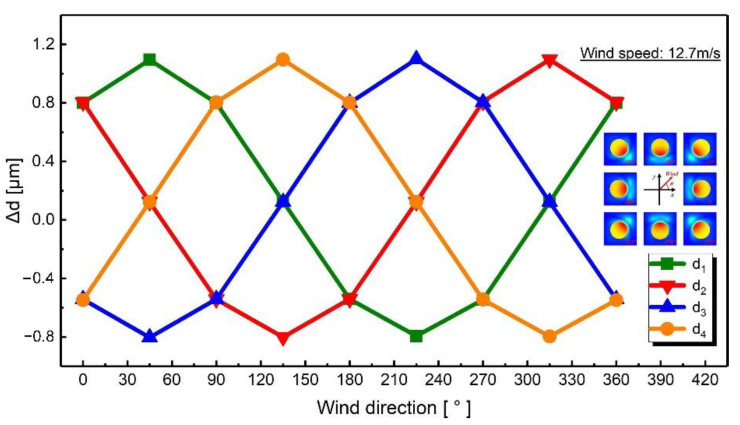
Simulated change in gap distance when the wind direction changed from 0° to 360° in the xy-plane with a fixed wind speed of 12.7 m/s.

In order to verify the feasibility of the proposed sensor, simulations were performed using the finite element method (FEM) solver (COMSOL 5.5). The *x* and *y* axes were equivalent due to the rotational symmetry of the sensor in the xy-plane. Therefore, the simulation variables included the wind speed in the xy-plane, the wind direction in the xy-plane, and the wind speed in the *z*-axis. The results of the simulations showed the change in the distance between the upper and lower electrodes, which characterized the deformation of the device and determined the change in four differential capacitances. Figure 3 shows the simulation results when the wind direction was fixed at 0° and the wind speed was changed from 0 m/s to 25 m/s in the xy-plane. Obviously, as the wind speed increased, the distances (d_1_ and d_2_) on the windward side increased and the distances (d_3_ and d_4_) on the leeward side decreased. Figure 4 shows the simulation results when the wind speed was fixed at 12.7 m/s and the wind direction was changed from 0° to 360° in the xy-plane. The distances between plates changed as a sine function to the wind direction.

**Figure 5:**
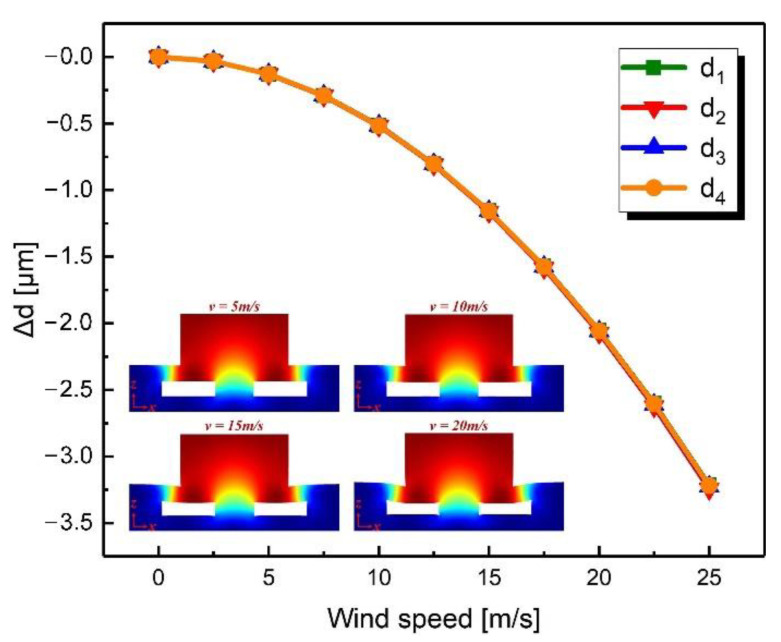
Simulated change in gap distance when the wind speed changed from 0 m/s to 25 m/s in the *z*-axis.

Figure 5 shows the simulation results when the wind speed was changed from 0 to 25 m/s in the *z*-axis. All distances decreased as the wind speed increased. The above simulation results are consistent with expectations. Moreover, it should be noted that at the same wind speed in the xy-plane the change in distance on the windward side was greater than that on the leeward side, which indicated that the deformation of the device caused by the tilt of the windward pillar was asymmetrical.

## 3. Fabrication

### 3.1. Material Selection

According to the operation principle, the material of the proposed flexible sensor should meet the special requirements. Firstly, the material should have plasticity to form a specific device structure. Secondly, the material should have a controllable Young’s modulus. The windward pillar and the support layer should have a large Young’s modulus to maintain the basic structure of the sensor, while the upper and lower electrode layers should have a small Young’s modulus to improve the sensitivity of the sensor. Thirdly, the material should have good adhesion with the metal to facilitate the preparation of the electrodes. Finally, the material should meet the bonding requirements so that all layers can bond together to form a complete device.

Based on the above considerations, PDMS was the most promising substrate material to fabricate the device. PDMS is prepared by mixing and curing the matrix and curing agent. Generally, the mass ratio of the matrix and curing agent has an influence on the Young’s modulus of the solidified PDMS. Table 2 shows the Young’s modulus of PDMS with different mass ratios [20], in which it can be seen that the higher the mass ratio is, the lower the Young’s modulus is. Therefore, the Young’s modulus of PDMS can be controlled by adjusting the mass ratio to meet the needs of different structural layers.

In addition, copper was chosen as the electrode material due to its good electrical conductivity, mature preparation technology, and low cost.

### 3.2. Fabrication Process

**Figure 6:**
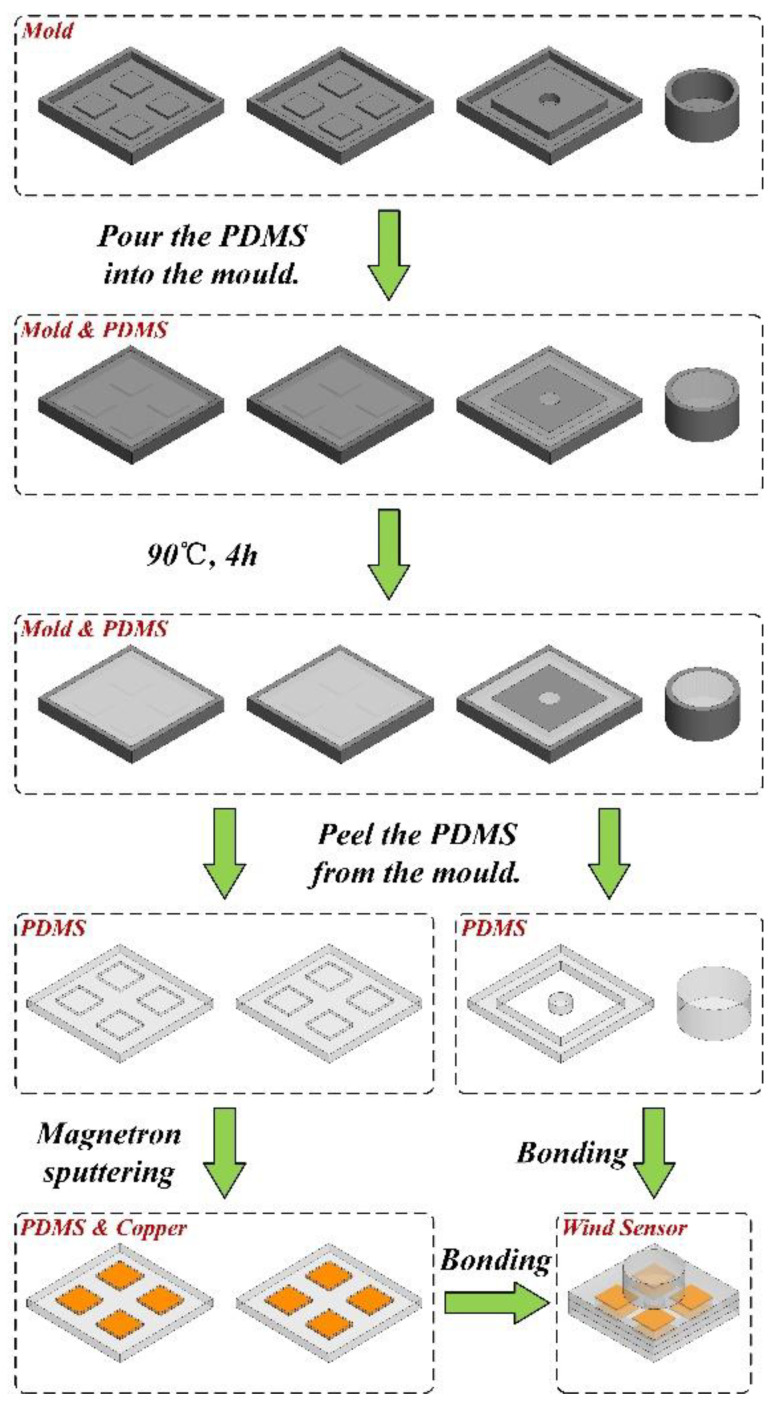
Fabrication process of the proposed flexible wind sensor.

The fabrication process of the proposed wind sensor is illustrated in Figure 6. Firstly, four molds corresponding to the structure layers were made using 3D printing technology. The matrix and curing agents of PDMS were then mixed in a mass ratio of 10:1 and 20:1. Subsequently, the former was poured into the mold corresponding to the windward pillar and the supporting layer, while the latter was poured into the mold corresponding to the upper and lower electrode layers. The PDMS and the mold were cured at a temperature of 90 °C for about 4 h. After that, the four structural layers were peeled from the mold. Next, copper was sputtered on the upper and lower electrode layers using magnetron sputtering technology, one of the physical vapor depositions (PVDs), to form the electrodes. Finally, the four structural layers were assembled into a complete device using plasma bonding technology.

**Figure 7:**
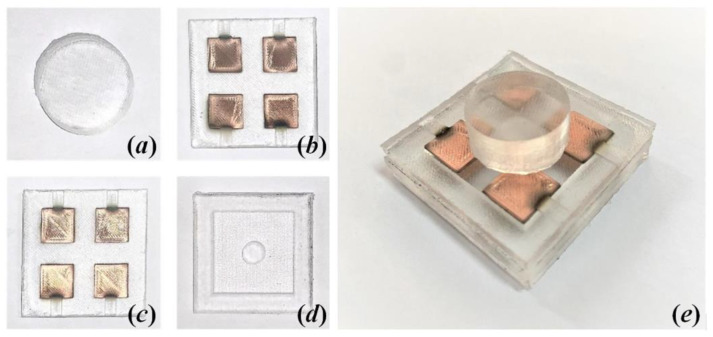
Photographs of (**a**) the windward pillar, (**b**) the upper electrode layer, (**c**) the support layer, (**d**) the lower electrode layer, and (**e**) the fabricated wind sensor.

Photographs of the fabricated wind sensor, as well as each layer, are shown in Figure 7. The dimensions of the sensor are close to 40 mm × 40 mm × 19 mm. Due to errors in the fabrication process, such as nonuniform gaps between copper electrodes, the four capacitors (C_1_, C_2_, C_3_ and C_4_) show different initial capacitances without external force, which are measured to be about 1.099, 1.085, 1.113, and 1.105 pF, respectively.

## 4. Measurements

**Figure 8:**
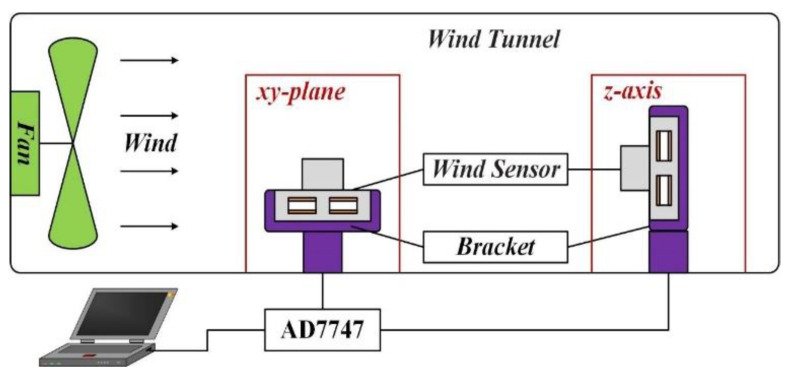
The setup for wind speed and direction measurement.

The setup for wind speed and direction measurement is shown in Figure 8 and it includes three parts: the wind speed experiments in the xy-plane, the wind direction experiments in the xy-plane, and the wind speed experiments in the *z*-axis. The sensor is fixed on the bracket placed in the wind tunnel. The wind speed is controlled by adjusting the fan speed, while the wind direction is controlled by rotating the bracket. The capacitances of the four capacitors are measured using an AD7747 evaluation board. In order to minimize errors in the measurements, the average value from 100 measurements was taken for every data point. In addition, since the initial capacitances of the four capacitors were different, the measured capacitances were not conducive to analysis and comparison. Therefore, it was necessary to normalize them (minus the initial capacitance) to get the capacitance change magnitude as the dependent variable of wind speed and direction.

### 4.1. Wind Speed Experiments in xy-Plane

**Figure 9:**
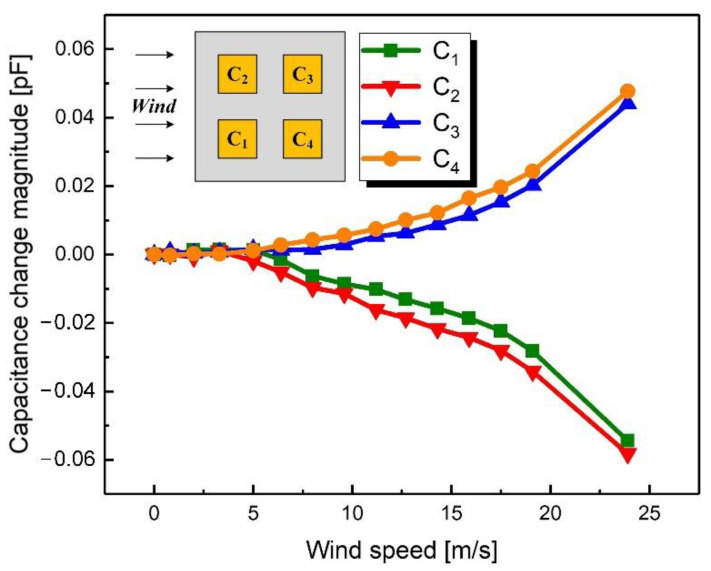
Measured capacitance change magnitudes when the wind speed varied from 0 m/s to 23.9 m/s with a fixed wind direction of 0°.

For these measurements, the wind direction was fixed at 0° (the direction of *x*-axis) and the wind speed was changed from 0 m/s to 23.9 m/s. Figure 9 shows the recorded capacitance change magnitudes as they changed with the wind speed. When the wind speed was between 0 m/s and 6.4 m/s, the capacitance did not change significantly (no more than 1 fF·m^−1^·s). When the wind speed was greater than 6.4 m/s, the wind would cause the effective deformation of the device. As the wind speed increased, C_1_ and C_2_ decreased while C_3_ and C_4_ increased, which was consistent with expectations. The maximum variation of capacitance was about 0.058 pF at a wind speed of 23.9 m/s, and the sensitivity of the device was 4 fF·m^−1^·s.

### 4.2. Wind Direction Experiments in xy-Plane

**Figure 10:**
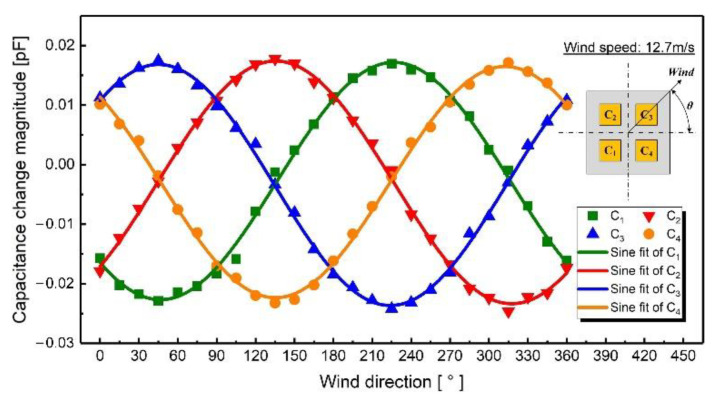
Measured capacitance normalized by the initial value when the wind speed was fixed at 12.7 m/s and the wind direction was changed from 0° to 360°.

For these measurements, the capacitance change magnitudes are shown in Figure 10, when the wind direction was changed from 0° to 360° with a fixed wind speed of 12.7 m/s. It can be deduced from the scatter diagram that the change trend of capacitances was similar to the trigonometric function, which is consistent with the simulations. Therefore, the sine function (y = y0 + Asin [ω(x-φ)]) is applied to fit the measured data, and the fitting results are shown in Table 3. The phase difference of adjacent capacitance change curves is about 90°. The extreme points of the capacitance change curves are 45°, 135°, 225°, and 315°, which were the diagonal directions of the device. The maximum variation of capacitance was up to 0.025 pF.

### 4.3. Wind Speed Experiments in z-Axis

**Figure 11:**
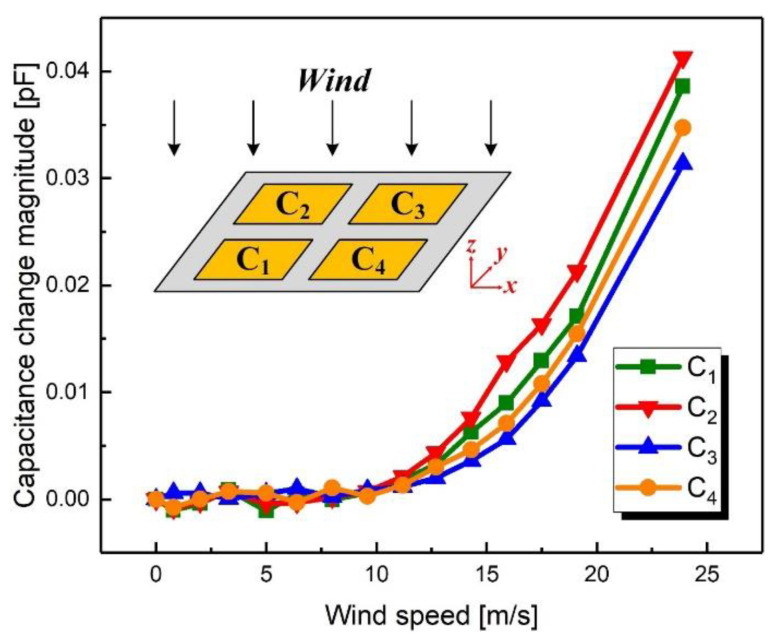
Measured capacitance change magnitudes when the speed was changed from 0 m/s to 23.9 m/s for the wind blowing from the positive *z*-axis to the negative *z*-axis.

For these measurements, the wind direction was fixed at the direction of the negative *z*-axis while the wind speed was changed from 0 m/s to 23.9 m/s. Figure 11 shows the relationship between the capacitance change magnitudes and the wind speed. It can be observed that when the wind speed was less than 9.6 m/s, the capacitance did not change significantly. When the wind speed was greater than 9.6 m/s, all four capacitances (C_1_, C_2_, C_3_ and C_4_) increased with the wind speed, which is consistent with expectations. The maximum variation of capacitance was 0.041 pF for the wind speed of 23.9 m/s and the sensitivity of the device was in the order of 3 fF·m^−1^·s.

## 5. Discussion

As shown in Figure 9 and Figure 11, the capacitances are not sensitive to the wind at low speeds. The reason is that the stress caused by the wind is proportional to the square of the speed. Therefore, at low wind speeds, the stress is too small to produce the deformation that can cause a change in capacitance. There are several solutions to improve the sensitivity of the device. On the one hand, the windward area of the device can be enlarged by increasing the height of the windward pillar so that the device is subjected to greater stress for the same wind speed. On the other hand, the Young’s modulus of the device may be reduced by increasing the proportion of the matrix in the PDMS so that the device undergoes greater deformation under the same stress. Additionally, trenches designed in the upper electrode layer can also change the gap distance to achieve improved sensitivity at low wind speeds. The maximum detectable wind speed is also higher than 23.9 m/s, but this was the limit of the wind tunnel used in this paper.

Moreover, the capacitance changes observed were inconsistent with the simulation results, which means that there were errors in the measurement results. First of all, the fringing field capacitance was not considered in the theoretical analysis. Secondly, the thickness of the structure layers was not uniform and the surface of the electrodes was uneven, leading to the differences in the performance of the four capacitors. Thirdly, the capacitance measurement results included the interference in the environment, which made the capacitance fluctuate. Finally, due to manual assembly, the center of the windward pillar was not strictly aligned with the center of the electrode layer, resulting in errors in the measurement results of the wind direction.

It should be noted that the proposed sensor cannot measure the speed for the wind from the negative direction of the *z*-axis. In order to remedy this defect, two 2.5D wind sensors could be superimposed to achieve the measurement of 3D wind.

## 6. Conclusions

In this paper, a novel flexible 2.5D wind sensor based on differential plate capacitors has been designed, simulated, fabricated, and characterized. The wind sensor can transform the 2.5D wind field information into capacitance information, where the wind direction is characterized by the change trend of capacitances and the wind speed is characterized by the change magnitude of capacitances. Moreover, the electrodes of the capacitors can be embedded in the substrate to avoid interference from the external environment, which would improve the reliability of the device. Experimental results demonstrate that the sensor can determine the wind direction over the full range of 360° in the xy-plane, and the sensitivities to wind speed are 4 fF·m^−1^·s and 3 fF·m^−1^·s in the xy-plane and *z*-axis, respectively. The proposed flexible wind sensor exhibits some potential in areas that require devices with low power consumption and high reliability.

## Figures and Tables

**Table 1:** Measurement principle along different axes.

Axes		*v* > 0	*v* < 0
*x*-axis	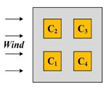	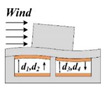	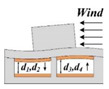
*y*-axis	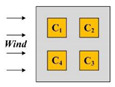	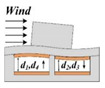	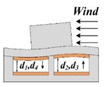
*z*-axis	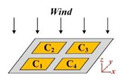	N/A	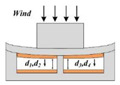

**Table 2:** Young’s modulus of solidified PDMS with different mass ratios.

Mass Ratio	Young’s Modulus [MPa]
8:1	2.15 ± 0.02
10:1	1.97 ± 0.03
12:1	1.62 ± 0.02
15:1	1.25 ± 0.03

**Table 3:** Fitting results of the measured capacitance change magnitude.

Parameters	C_1_	C_2_	C_3_	C_4_
y_0_	−0.00276	−0.00298	−0.00342	−0.00293
A	0.01993	0.02038	0.02024	0.01942
ω	1.00065	0.98817	1.00662	1.01593
φ	136.6596	44.39655	−44.23719	−136.10507

## Data Availability

The data presented in this study are available on request from the corresponding author.
